# A Comparison of Grizzly Bear Demographic Parameters Estimated from Non-Spatial and Spatial Open Population Capture-Recapture Models

**DOI:** 10.1371/journal.pone.0134446

**Published:** 2015-07-31

**Authors:** Jesse Whittington, Michael A. Sawaya

**Affiliations:** 1 Parks Canada, Banff National Park Resource Conservation, Banff, Alberta, Canada; 2 Sinopah Wildlife Research Associates, Missoula, Montana, United States of America; University of Alberta, CANADA

## Abstract

Capture-recapture studies are frequently used to monitor the status and trends of wildlife populations. Detection histories from individual animals are used to estimate probability of detection and abundance or density. The accuracy of abundance and density estimates depends on the ability to model factors affecting detection probability. Non-spatial capture-recapture models have recently evolved into spatial capture-recapture models that directly include the effect of distances between an animal’s home range centre and trap locations on detection probability. Most studies comparing non-spatial and spatial capture-recapture biases focussed on single year models and no studies have compared the accuracy of demographic parameter estimates from open population models. We applied open population non-spatial and spatial capture-recapture models to three years of grizzly bear DNA-based data from Banff National Park and simulated data sets. The two models produced similar estimates of grizzly bear apparent survival, per capita recruitment, and population growth rates but the spatial capture-recapture models had better fit. Simulations showed that spatial capture-recapture models produced more accurate parameter estimates with better credible interval coverage than non-spatial capture-recapture models. Non-spatial capture-recapture models produced negatively biased estimates of apparent survival and positively biased estimates of per capita recruitment. The spatial capture-recapture grizzly bear population growth rates and 95% highest posterior density averaged across the three years were 0.925 (0.786–1.071) for females, 0.844 (0.703–0.975) for males, and 0.882 (0.779–0.981) for females and males combined. The non-spatial capture-recapture population growth rates were 0.894 (0.758–1.024) for females, 0.825 (0.700–0.948) for males, and 0.863 (0.771–0.957) for both sexes. The combination of low densities, low reproductive rates, and predominantly negative population growth rates suggest that Banff National Park’s population of grizzly bears requires continued conservation-oriented management actions.

## Introduction

Increasing human activity throughout the world threatens many species and subsequent ecosystem processes [1]. Basic metrics such as population growth rates are required to help understand how human activities, ecological conditions, and management actions affect the conservation status of wildlife populations. Capture-recapture techniques are commonly used to estimate abundance, density, and demographic parameters such as population growth, apparent survival, and recruitment. Capture-recapture studies use repeated surveys of identifiable individuals to estimate detection probability and variance around density, apparent survival, recruitment, and population growth rates [2].

Within closed population capture-recapture studies, multiple sampling occasions generate individual capture histories that are then used to estimate detection probability and the number of individuals in the study area that were present but undetected. When surveys are conducted across multiple years or sessions, open population capture-recapture models track individual detections over time to estimate demographic parameters such as apparent survival, per capita recruitment, and population growth rates [3,4]. In its simplest form, non-spatial capture-recapture models determine whether or not each animal was detected within an occasion and use the proportion of occasions each animal was detected to estimate detection probability. Challenges with capture-recapture arise when individuals vary in their exposure to traps. For example, animals with home ranges that occur entirely within a study area may have higher detection probabilities than animals with home ranges that only partially overlap the study area. This variability in detection probability is pronounced for wide ranging carnivores that have large home ranges relative to the size of the study area. Capture-recapture models have included the distance between an animal’s home range center and the edge of the study area as a covariate affecting detection probability [5–8], but this approach assumes a linear relationship between distance to edge and detection probability and does not reflect observation processes. Spatial capture-recapture techniques are a rapidly evolving class of models that directly estimate the effects of distance between an animal’s home range centre and each trap location on probability of detection [9–12].

Comparisons between closed population non-spatial and spatial capture-recapture models have found that spatial models generally provide more robust density estimates with fewer biases [13–16] but can be biased low [17]. Spatial capture-recapture methods have been used to estimate densities of many species including lynx [16], wolverine [18], and black bears [19–21]. However, most studies have focussed on single year models and only a few studies have used open population spatial capture-recapture approaches to estimate population parameters [12,22–24]. Comparisons between open population non-spatial and spatial models found via simulation that the non-spatial models under-estimated mortality rates [23]. No studies to our knowledge have compared population growth rates, apparent survival, and per capita recruitment using non-spatial and spatial capture-recapture models.

DNA-based capture-recapture has been used extensively to estimate the abundance of bears [6,8,20,25–28] and many other wildlife species. Grizzly bear (*Ursus arctos*) populations are vulnerable to population declines because they occur in low densities, have large home ranges, and low reproductive rates. Human developments affect grizzly bears in many ways. At a large scale, they fragment and isolate populations of grizzly bears [29]. Within subpopulations, grizzly bear mortality rates increase in areas with high road density and near human developments [30–33]. Causes of mortality near human developments include removal or euthanization from human-bear conflicts, legal or illegal harvest, and collisions with vehicles or trains. Further expansion of human developments would likely reduce survival rates and some source populations could become sink populations [34]. Given these threats, monitoring changes in population parameters is important for the conservation and management of grizzly bears.

Our objectives were to compare per capita recruitment, apparent survival, and population growth rates estimated from open population non-spatial and spatial capture-recapture models with grizzly bear and simulated capture-recapture data. We compared demographic parameters using three years of data from a grizzly bear population in Banff National Park (hereafter referred to as Banff) and data simulated across a range of detection probabilities and densities.

## Methods

Our research did not involve capture or handling of animals, therefore did not require approval of animal care and use procedures. Permissions for field studies in BNP were given by Parks Canada Agency under research permit BAN-2007-999. Permissions for field studies in Alberta provincial lands were given by the Alberta Minister of Community Development under research permit RC-06-22.

### Study Area

Our study area encompassed 2246 km^2^ of Banff National Park, Alberta, Canada (51.2° N, 115.5° W). Banff received over 3 million visitors per year [35]. Banff contained rugged topography, short summers, and long cold winters [36] and was located on the eastern and generally lee side of the Continental Divide. Forests were dominated by Engelmann spruce (*Picea engelmanii*) and subalpine fir (*Abies lasiocarpa*) in the subalpine and lodge-pole pine (*Pinus contorta*) in the Montane. Carnivores in the region included wolverine (*Gulo gulo*), lynx (*Lynx canadensis*), cougar (*Puma concolor*), red fox (*Vulpes vulpes*), coyote (*Canis latrans*), wolf (*Canis lupus*), black bears (*Ursus americanus*), and grizzly bears. Ungulates in Banff included elk (*Cervus Canadensis*), moose (*Alces alces*), mule deer (*Odocoileus hemionus*), white-tailed deer (*O*. *virginianus*), bighorn sheep (*Ovis Canadensis*), and mountain goat (*Oreamnos americanus*).

### Data Collection

We used data from a grizzly bear DNA-based capture-recapture study conducted from 2006 to 2008 to evaluate the effectiveness of wildlife crossing structures [6,37,38]. Hair samples were collected from barbed wire set up at three types of hair capture stations: hair traps with liquid scent lure [26], bear rubs with no lure [27], and wildlife crossing structures with no lure [39]. Timing of sampling effort and trap deployment is provided in S1 Fig. Detailed descriptions of the sampling methods, DNA extraction methods, and capture-recapture results can be found in Sawaya et al. [6]. We omitted bear rubs that were sampled but failed to collect hair samples. All capture locations encompassed 2246 km^2^ centred on the Trans-Canada highway. GPS collared female and male grizzly bears in the area had average 95% minimum convex polygon home ranges of 407 km^2^ (n = 10) and 1140 km^2^ (n = 11) respectively. We sampled 42 7x7 km grid cells with hair traps in 2006 and 2008. We set up one hair trap per cell and moved them every two weeks for five occasions spread across the spring and summer. Twenty wildlife crossing structures were sampled continuously for the duration of the study with eight occasions per year. Bear rubs were sampled the latter half of 2006 and throughout 2007 and 2008. We sampled 284, 321, and 313 bear rubs during 2006, 2007, and 2008 respectively for up to seven occasions per year. We used the 192 bear rubs that detected bears in the analysis. During 2006, we extracted DNA from all hair samples. We sub-sampled hair traps during 2008 and bear rubs during 2007 and 2008 [6].

### Statistical Analysis

We calculated open population non-spatial and spatial capture-recapture demographic rates using Markov chain Monte Carlo (MCMC) methods. For each method we estimated the number of individuals alive (*N*) during each year, per capita recruitment (*R*), apparent survival rate (ϕ), and the population growth rate (λ) using a data augmentation approach [12,22,40,41]. We determined whether or not each individual bear *i* was detected at trap *j* during sampling occasion *k* in year *t*. We augmented the population of detected animals by 200, 200, and 280 undetected animals for the female, male, and combined sex models respectively. We then used the latent state variable *z*_*it*_ to indicate whether individual *i* was alive (*z* = 1) or dead (*z* = 0) during each year. We used the state of *z*_*it*_ to determine annual abundance in the state-space, density, per capita recruitment, apparent survival, and population growth. We applied these data augmentation methods to non-spatial (CR_*dedge*_) and spatial (SCR) models. Both models included sex, trap type, and occasion specific estimates of detection probability. We ran CR_*dedge*_ and SCR models for females only, males only, and combined females and males.

#### Non-spatial capture-recapture

Non-spatial capture-recapture analyses conceptually modelled detection probability as the proportion of sampling occasions individuals were detected. Our capture-recapture model (CR_*dedge*_) modelled the detection history of individual *i* during sampling occasion *k* at trap type *m* in year *t* using (*y*_*ikm*_ | *z*_*it*_ = 1) ~ *Bernoulli*(*p*_*km*_). We calculated separate likelihoods and thus separate detection probabilities for each type of trap [42]. We used a proportional hazard approach to model the effects of survey effort on detection probability [43]. Thus, $p_{effort\;kmt}\;=\;1-{\left(1-p_{km}\right)}^{{NDays}_{kmt}/{ReferenceDays}_{m}}$ where *p*_*km*_ is the baseline detection probability for trap type *m* on occasion *k*, *NDays*_*kmt*_ was the total number of trap days for trap type *m* during occasion *k* and year *t*, and *ReferenceDays*_*m*_ was the median number of active trap days for trap type *m*, which was 162, 42, and 20 days for bear rubs, hair traps, and highway crossings respectively. Consequently, probability of detection was scaled such that it equalled 0 when *NDays* was 0 and approached 1 as *NDays* approached infinity. Trap specific detection probability varied within a year by sampling occasion. Variability in detection probability among years depended on the number of active traps. The cumulative probability of detection within sampling occasion *k* and year *t* was 1 –(1 –*p*_*effort Bear Rub kt*_) (1 –*p*_*effort HairTrap kt*_) (1 –*p*_*effort Highway Crossing kt*_). Bears with home range centres near the edge of the study area were exposed to fewer traps and thus had lower detection probabilities [5–7]. For each individual detected, we calculated its home range centre as the centroid of a 100% minimum convex polygon around traps where the bear was detected. We used the trap location or the average of two locations for bears detected at one and two traps respectively. We calculated the distance between the home range centre and the edge of the study area (DEDGE; km). We modeled probability of detection as a function of trap type *m*, occasion *k*, and DEDGE where *logit*(*p*_*ikm*_) = *B*_*mk*_+ *B*_DistEdge_*DEDGE_*i*_. For our combined sex model, detection probability varied by sex, trap type, and occasion. The augmented population of bears lacked an empirical measure for distance to edge. For these bears we generated values for DEDGE within the MCMC model using a Gamma(*shape*_*dedge*_, *rate*_*dedge*_) distribution with the parameters *shape*_*dedge*_ and *rate*_*dedge*_ estimated from the observed bears. The gamma distribution was a positive continuous distribution with skewness driven by *shape*_*dedge*_. We truncated simulated values of DEDGE at 18.5 km which was the maximum observed value of DEDGE from detected bears.

#### Spatial capture-recapture

Spatial capture-recapture analyses essentially modelled detection probability as the probability of detecting an individual at a single trap during a single sampling occasion. Detection probability was scaled by the distance between the trap and the individual’s home range centre. Our spatial capture-recapture models (SCR) built upon closed population models of black bears [20] and open-population MCMC models from camera-trap studies [22]. We determined whether each individual *i* was detected at trap *j* on occasion *k* during year *t* and modelled probability of detection *p*_*ijk*_ as (*y*_*ijk*_ | *z*_*it*_ = 1) ~ *Bernoulli* (*p*_*ijk*_). We used a bivariate (half) normal detection function where probability of detection *p*_*ijk*_ depended on the probability of captures at the home range center (ɡ_*0*_), distance (*D*_*ij*_) between the estimated home range centre *s*_*i*_ trap *j*, and the home range size scale parameter (σ) such that $p_{ij}\;=\;g_{0}∙exp(\frac{{-D}_{ij}^{2}}{2\;∙\;\sigma^{2}})$. We included the effects of trap type *m* and occasion *k* on ɡ_*0*_ was using *logit*(*g*_0__*km*_) = *β*_*km*_. Detection probability for the combined sex model varied by sex, trap type, and occasion. Home range centres were unobserved latent variables that could occur anywhere in the state-space. We defined the state space as a 25 km buffer around all trap locations.

#### Open population parameters

During each iteration of the MCMC sampling, each individual *i* was classified according to the latent state variable as alive (*z*_*it*_ = 1) or dead (*z*_*it*_ = 0) during year *t* [22]. For individuals alive in year *t*−1, their probability of being alive in year *t* was the apparent survival rate ϕ_*t*_. Individuals that had never been alive at year *t*−1 had a probability γ_t_ of being recruited into the population. Together, the latent state for an animal being alive in year *t* was *z*_*it*_ ~ *Bernoulli*(*ϕ*_*t*_ *z*_*i,t*−1_+ *γ*_*t*_ [1− *z*_*i,t*−1_]). We calculated per capita recruitment *R* as the number of new individuals at time *t* divided by the number of animals alive at time *t*−1. Population growth rate (λ_*t*_) was a derived parameter, which we calculated as ϕ_*t*_ + *R*_*t*_ [40]. We calculated the average estimates of ϕ, *R*, and λ as the geometric mean of year 1 and 2 estimates for each MCMC iteration.

We assessed model fit using a Bayesian *P*-value goodness of fit test by comparing summed observed and predicted Freeman-Tukey residuals [12]. Bayesian *P*-values were calculated as Pr(χ^2^_*observed*_ > χ^2^_*simulated*_) and values less than 0.05 or greater than 0.95 indicated lack of fit.

We used non-informative priors for all parameters. Priors from the CR_*dedge*_ models: β_*TrapType*_ and β_*DistEdge*_ were Uniform(-10, 10); and shape_*dedge*_ and rate_*dedge*_ Uniform(0, 30). Priors from the SCR models: σ was Uniform(0, 15); β_*TrapType*_ was Uniform(-10, 10); and home range centres had Uniform(-56, 56) and Uniform(-50, 50) for east-west and north-south coordinates respectively. Priors for both CR_*dedge*_ and SCR: probability of *z*_*i*_ being a male (*p*_*male*_), ϕ_*t*_, and γ_*t*_ had Uniform(0, 1). Interactions between individual *i* being a male and covariates for detection probability had Uniform(-10, 10) priors. We ran MCMC models with 3 chains, and 30 000 iterations that followed a burn in period of 5000 discarded iterations. We assessed MCMC convergence by examining traceplots and the Gelman–Rubin statistic where values < 1.1 suggested convergence [40].

We ran all analyses using R version 3.1.3 [44] and the package jagsUI 1.3.1 [45] to access JAGS 3.4.0 [46]. We provided our grizzly bear data as well as our combined sex open CR_*dedge*_ and SCR population models in S1 Data and S1 Text.

### Simulation Study

We used simulations to compare relative bias, power, and credible interval coverage of non-spatial and spatial capture-recapture models similar to Gardner et al. [22] and Efford et al. [43]. We simulated three years of capture-recapture data from a square grid of 10 x 10 traps with trap spacing *s* equal to σ, which we set to 1.0 km. We generated a fixed density of home range centres randomly within the study area which we defined as 2.5 *s* buffer around trap locations. We used densities of 0.5 and 1.0 *s* ^-2^. Each individual alive at year *t* had a ϕ = 0.8 probability of survival to *t* + 1. Each individual alive at year *t* also had an *R* = 0.1 probability of introducing one new animal anywhere within the study area in year *t* + 1. Population growth rate (λ) was ϕ + *R* = 0.9. The initial population size was fixed and the subsequent population sizes were random variables.

We generated detection histories with five sampling occasions per year. Each individual had an independent probability of detection at each trap based on the distance between the trap, its home range centre, and a half normal detection function. We simulated capture histories using ɡ_*0*_ = 0.1 and 0.5. Values used in simulations, which were similar to Efford [43], were selected with an intent to reflect real-world ecological systems with both low and high cumulative probability of detection, roughly 50 to 200 individuals detected, and population declines of 10% per year which many land managers would hope to detect. For each simulated data set we ran a capture-recapture model with no covariates for detection probability (CR), a capture-recapture model with distance between the observed home range centre and the edge of the study area as a covariate affecting detection probability (CR_*dedge*_), and a spatial-capture recapture (SCR) model. Models included annual estimates of ϕ_*t*_ and γ_*t*_.

We ran open population MCMC models using 3 chains, a burnin of 4000 iterations and repeating cycles of 5000 iterations until Gelman-Rubin convergence statistics for all parameters were less than 1.1. We simulated 100 data set per scenario and used the same simulated data sets for CR, CR_*dedge*_, and SCR. We compared the posterior median and the 95^th^ highest posterior density (HPD) interval results to values of ϕ and *R* used to simulate data. We calculated bias as the difference between each simulated posterior median and the true values. We calculated credible interval coverage as the percentage of simulations where the 95% HPD included the true value used to simulate the data. We calculated statistical power as the percentage of simulations where the upper 95% HPD limit for population growth was less than 1.0 [47]. R scripts used to run simulations are provided in S2 Text.

## Results

### Grizzly Bear Demographics

We detected 80 grizzly bears from 2006 through 2008 (Table 1). Bear rubs detected more individuals, had higher recapture rates, and had more active traps than hair traps or highway crossing structures. Re-capture rates increased from 2006 to 2008 and one bear was captured 50 times during the three years. We detected 3 and 5 new females and 10 and 5 new males in 2007 and 2008 respectively. Five grizzly bears died of human caused mortality in the study area from 2006 through 2008 including one male, one female, and three juveniles.

**Table 1:** Number of individual grizzly bears detected by trap type and year, mean number of detections per animal per year, and percent animals with greater than one detection per year.

TrapType	Number Individuals	Number Female	Number Male	Mean Detections per Year	Percent with > 1 Detection
2006	57	25	32	3.4	63.2
2007	49	18	31	5.4	69.4
2008	48	22	26	6.5	85.4
Bear Rub	73	29	44	3.4	57.8
Hair Trap	42	22	20	1.2	34.3
Highway Crossing	15	7	8	0.7	15.6
Total	80	33	47	5.0	72.1

**Fig 1:**
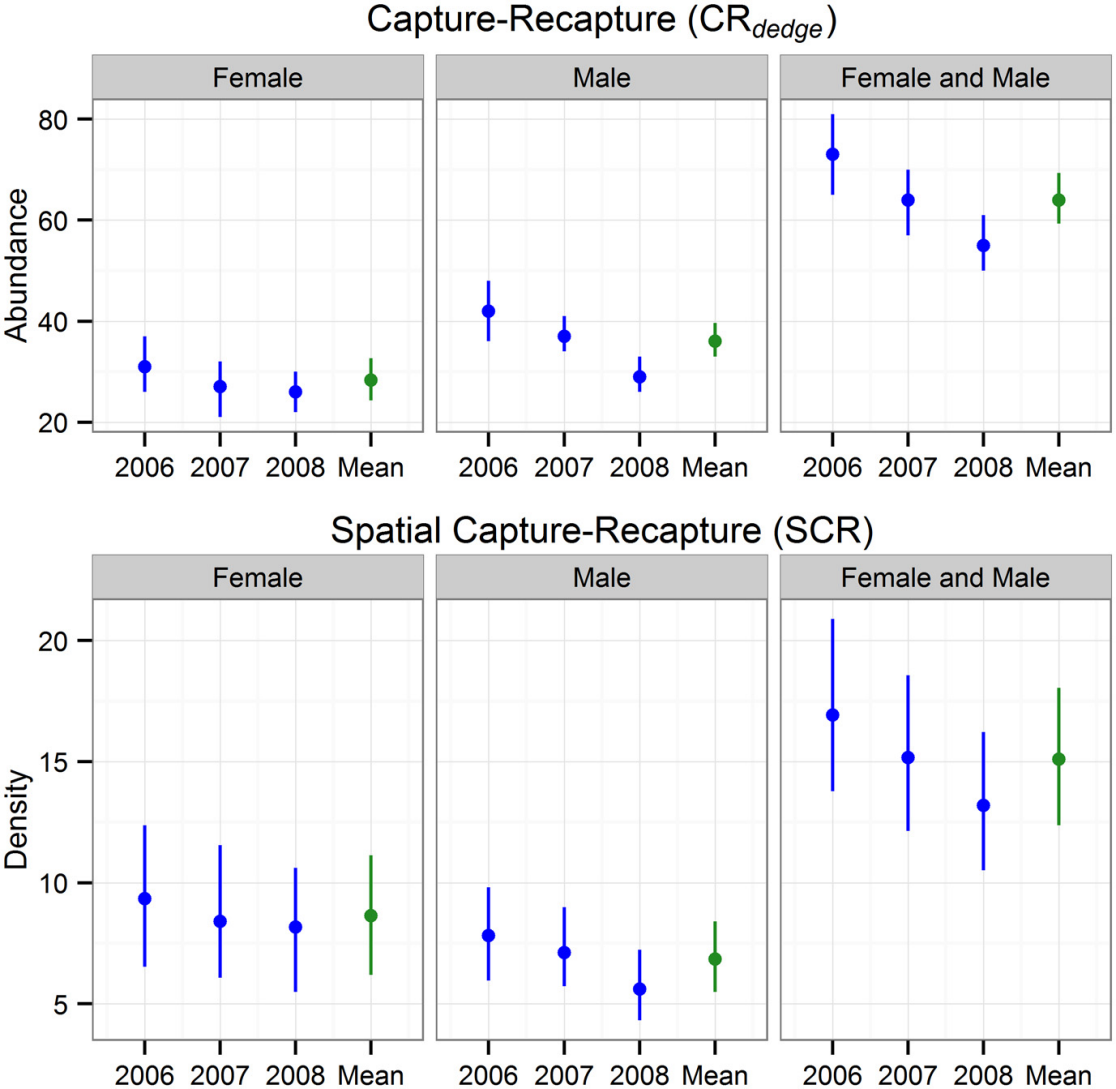
Grizzly bear abundance and density posterior medians with 95% HPD credible intervals for non-spatial (CR_*dedge*_) and spatial (SCR) capture-recapture models.

**Fig 2:**
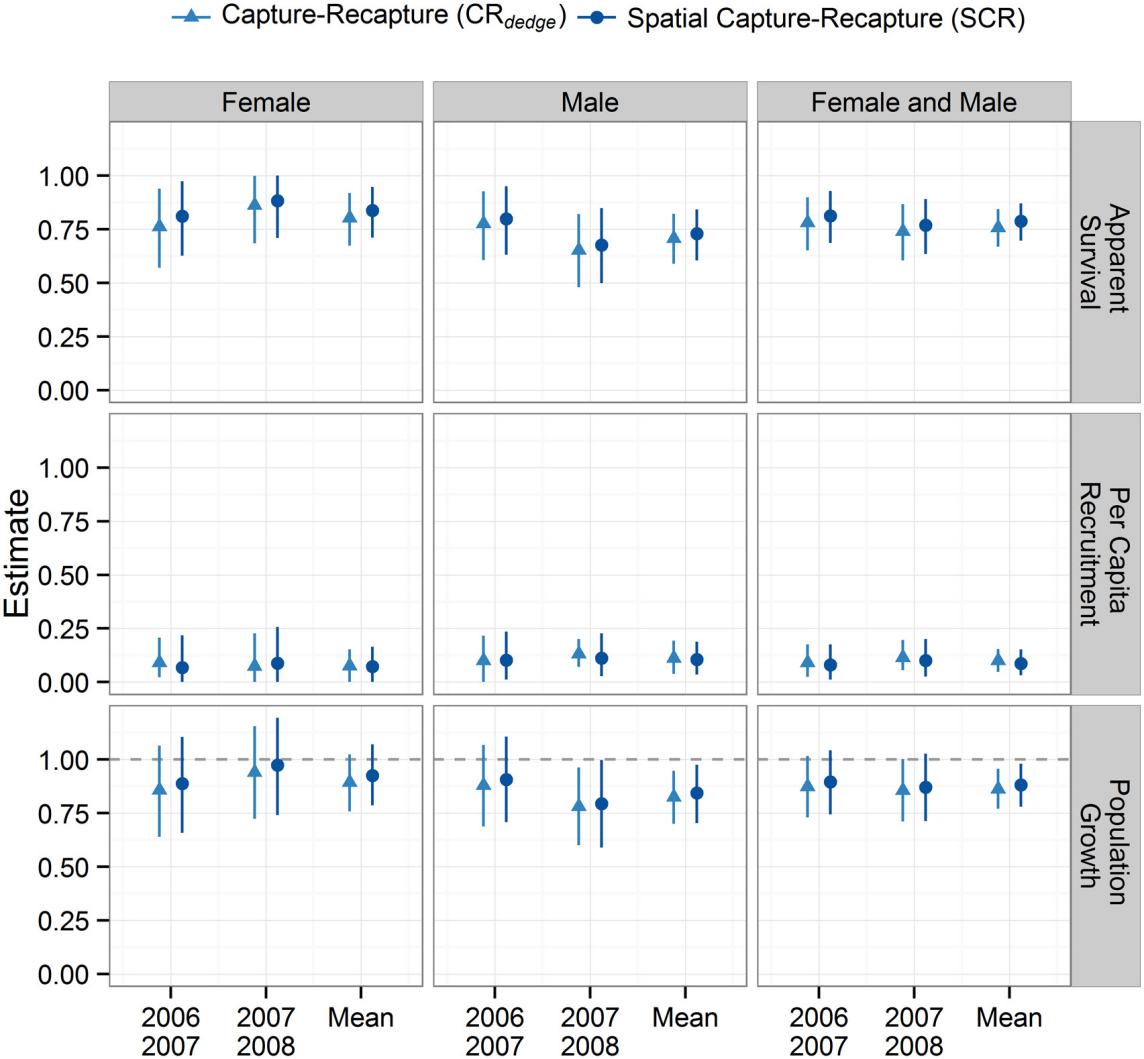
Grizzly bear median apparent survival, per capita recruitment, and population growth rates and 95% HPD credible intervals for non-spatial (CR_*dedge*_) and spatial (SCR) open population models. Dashed line for population growth rate equal to one indicates a stable population.

The open population SCR models had much better fit than the CR_*dedge*_ models (Table 2). The two classes of models produced similar estimates of population growth, apparent survival, and per capita recruitment (Figs 1 and 2). Both models suggested a population decline in the male segment of the population. Differences between SCR and CR_*dedge*_ point estimates were very small: 0.029, -0.008, and 0.023 for apparent survival, per capita recruitment, and population growth respectively. The difference in point estimates divided by SCR estimates ranged between 2.6 and 9.9%. Differences in the width of 95% HPD intervals were also very small: -0.008, 0.031, and, 0.024 for apparent survival, per capita recruitment, and population growth respectively. The coefficients of variation for abundance and density were less than 16% for all models and was generally lower for the CR_*dedge*_ models.

**Table 2:** Parameter estimates averaged from 2006–2008, 95% HPD intervals, and coefficient of variation from non-spatial (CR_*dedge*_) and spatial capture-recapture (SCR) models for female, male, and combined sex models. Annual estimates of population parameters are provided in S1 Table.

Model	Sex	Parameter	Median	HPD95_lower_	HPD95_upper_	CV
**CR**_***dedge***_	Female	λ	0.894	0.758	1.024	
		N	28.000	24.000	33.000	7.8
		*R*	0.076	0.000	0.152	
		φ	0.803	0.673	0.920	
		Model Fit	0.900	-	-	
	Male	λ	0.825	0.700	0.948	
		N	36.000	33.000	40.000	4.8
		*R*	0.112	0.039	0.193	
		φ	0.708	0.590	0.822	
		Model Fit	0.920	-	-	
	Female & Male	λ	0.863	0.771	0.957	
		N	64.000	59.000	69.000	4.1
		*R*	0.100	0.047	0.153	
		φ	0.757	0.669	0.844	
		Model Fit	0.970	-	-	
**SCR**	Female	D	8.638	6.187	11.128	14.6
		λ	0.925	0.786	1.071	
		*R*	0.072	0.000	0.165	
		φ	0.837	0.712	0.948	
		σ	4.988	4.626	5.397	
		Model Fit	0.520	-	-	
	Male	D	6.848	5.486	8.405	11.1
		λ	0.844	0.703	0.975	
		*R*	0.105	0.035	0.187	
		φ	0.730	0.606	0.843	
		σ	8.923	8.295	9.630	
		Model Fit	0.450	-	-	
	Female & Male	D	15.097	12.373	18.054	9.6
		λ	0.882	0.779	0.981	
		*R*	0.087	0.032	0.152	
		φ	0.787	0.697	0.871	
		σ female	4.993	4.615	5.382	
		σ male	8.964	8.309	9.641	
		Model Fit	0.470	-	-	

Parameter descriptions: φ = apparent survival, *R* = per capita recruitment, λ = population growth rate, N = number of individuals, D = density per 1000 km^2^, σ = the scale parameter for detection probability, Model Fit = Bayesian P-value where values < 0.05 or > 0.95 indicate poor fit.

**Fig 3:**
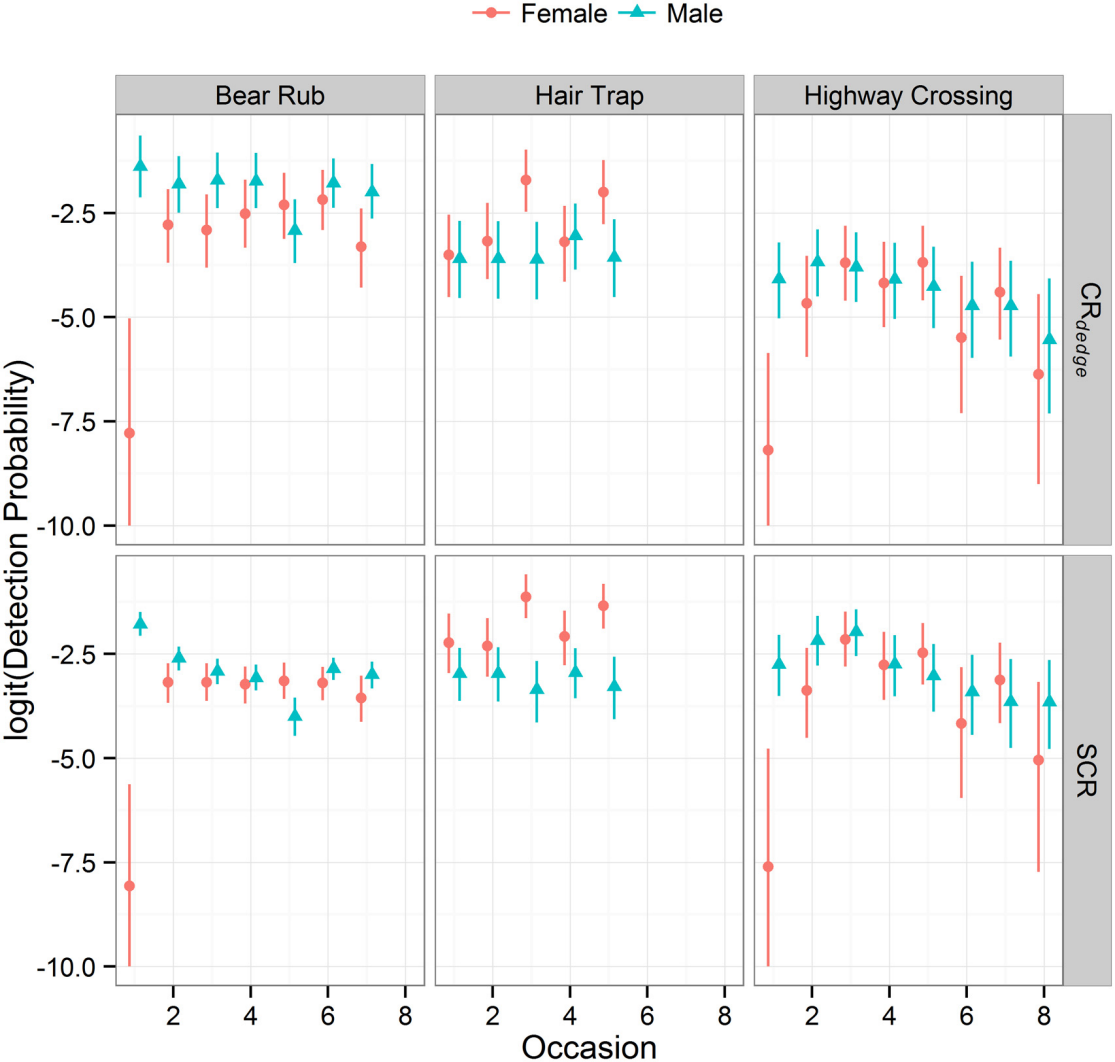
Occasion specific detection probability for female and male grizzly bears. Values for non-spatial capture-recapture models (CR_*dedge*_) were based on 162, 42, and 20 active traps for bear rubs, hair traps, and highway crossings respectively. Values for spatial capture-recapture (SCR) models indicate individual detection probability at a single trap in the middle of an individual’s home range centre. Detection probability varied by sex, trap type, and sampling occasion with a year. Variability in detection probability among years depended on the number of active traps (CR_*dedge*_ and SCR) and the distribution of traps (SCR).

Detection probability was highest in early summer for both sexes (Fig 3). Detection probability was low for females in the early spring and late fall. SCR model results showed that females had higher detection probability at hair traps than bear rubs. The cumulative probability of detection reflected in the CR_*dedge*_ detection probabilities was slightly higher for males at bear rubs than hair traps. Females had similar cumulative probability of detection at bear rubs and hair traps. Plots for the posterior distribution of home range centres show that detections were clustered in several regions of the study area and especially the Cascade Valley (S2 Fig). All parameters had Gelman-Rubin statistics ≤ 1.01. Estimates for all model parameters can be found in S1 Table.

### Simulation Study

**Fig 4:**
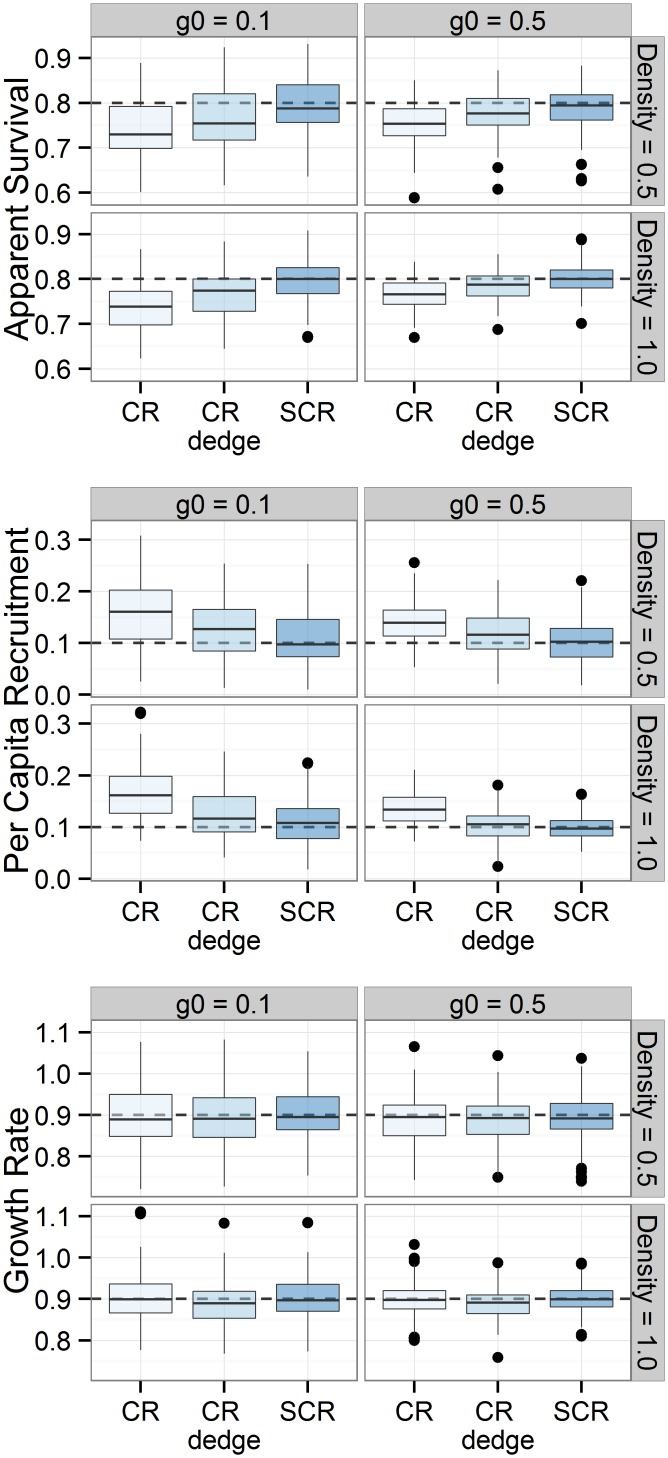
Boxplots showing the range of apparent survival, per capita recruitment, and population growth rate posterior medians generated from 100 simulated data sets per scenario. Dashed lines indicate true values used to simulate the capture-recapture data.

**Fig 5:**
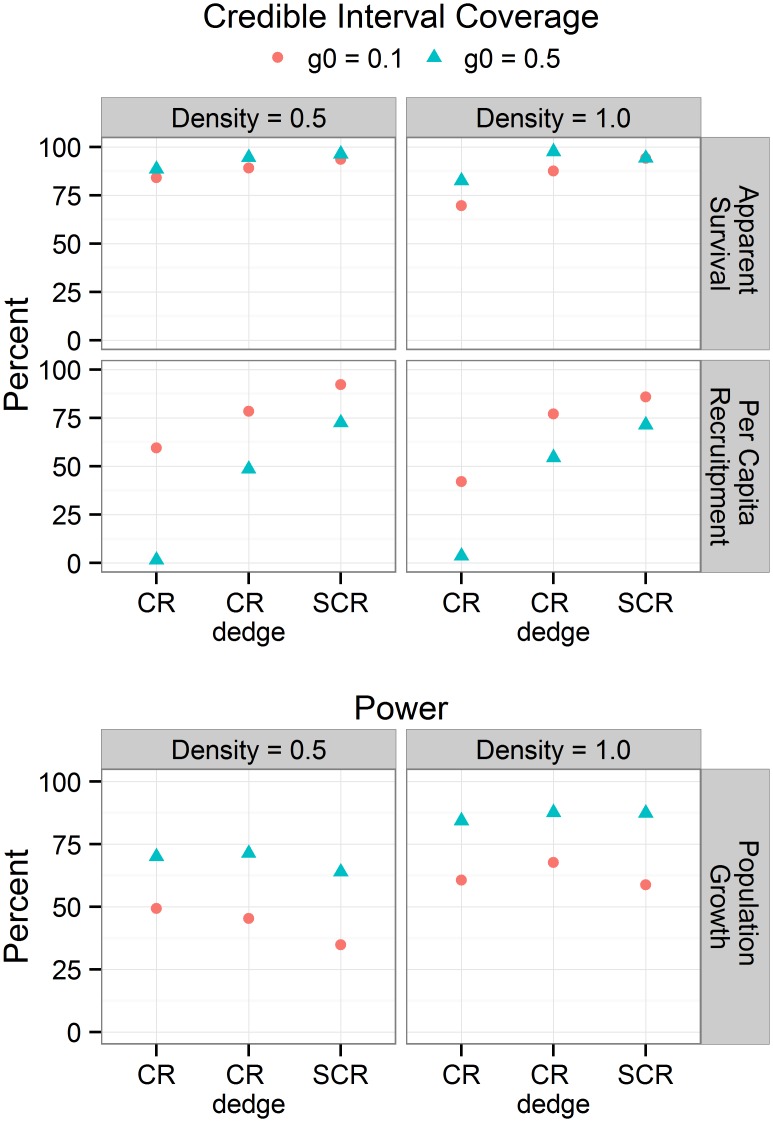
Percent of simulations where the 95% HPD encompassed the true value (credible interval coverage) and percent of simulations where the upper 95% HPD was less than 1.0 (power).

The average number of individuals detected in our simulations ranged from 86 for low density (D = 0.5 individuals km^-2^) and low detection probability (*g*0 = 0.05) to 211 for high density (D = 0.5 individuals km^-2^) and high detection probability (*g*0 = 0.5). SCR models produced consistently more accurate estimates of apparent survival and per capita recruitment than CR and CR_*dedge*_ models (Fig 4). Including DEDGE as a covariate affecting capture probability increased the accuracy of non-spatial capture-recapture models. Increasing detection probability and density resulted in decreased bias and increased accuracy. The maximum average bias between posterior medians and truth for apparent survival and per capita recruitment was 0.06, 0.03, and 0.01 for CR, CR_*dedge*_, and SCR respectively (S2 Table). Most non-spatial models exhibited negative biases for apparent survival and positive biases for per capita recruitment. These biases offset each other to produce derived growth rates with minimal bias. SCR models had higher credible interval coverage than CR and CR_*dedge*_ models (Fig 5). The three methods had similar power to detect significant population trends at high density and detection probability. However, SCR had slightly lower power to detect trends under scenarios with low density and low detection probability. CR and CR_*dedge*_ exhibited high Type I error (low CIC) in these scenarios. The Gelman-Rubin statistic for convergence was less than 1.05 for all parameters in all simulations. See S2 Table for complete simulation results.

## Discussion

A growing number of studies have shown that spatial capture-recapture models outperform non-spatial models in terms of accuracy when producing annual density estimates [13–16,23] but see Gerber and Parmenter [17]. However, one of the objectives of long-term monitoring programs is to determine how populations change over time. No studies to our knowledge have directly compared demographic rates from non-spatial and spatial capture-recapture models. Our simulation results suggest that open population spatial models have fewer biases and better credible interval coverage than non-spatial models, although there was little difference between empirical results. Choosing to use non-spatial models can lead to negatively biased estimates of apparent survival and positively biased estimates of per capita recruitment, especially in study areas with low density and low detection probability. The likely reason non-spatial models had negatively biased apparent survival is that animals on the periphery of the study area were detected in the first year but not the second or third year even though they were still alive and did not emigrate. Conversely, per capita growth rates were positively biased because animals that were alive and present in year 1 were not detected until year 2 or 3. Biases in these parameters can lead to incorrect inference and biases in derived parameters such as population growth rates. Interestingly, both Gardner et al. [22] and our study found slightly negative biases in apparent survival. Including covariates for distance to edge in non-spatial models substantially improved the accuracy of estimates of apparent survival and per capita recruitment similar to improvements in estimates of annual population abundance found in other studies [5–7].

Our grizzly bear CR_*dedge*_ and SCR estimates and credible intervals of all demographic parameters were similar. This was somewhat surprising given our low density population of grizzly bears, relatively small study area size, and the biases associated with CR_*dedge*_ at low densities. One possible reason for the similarity in estimates is that we maximized the cumulative probability of detection with a large number of detectors and our simulations showed accuracy improved with detection probability. We also included distance to edge as a covariate for detection probability and used multiple detector types to reduce bias [6,28]. The convergent CR_*dedge*_ and SCR estimates for a slightly negative grizzly bear population trajectory increases our confidence in these results. The overall decline in grizzly bear density was driven more by changes in male than female density, which could be expected given the wide-ranging nature of males. The 95% credible intervals for females but not males encompassed the value 1.0 for a stable population. Similar to Garshelis et al. [48] males in our study area had lower apparent survival rates than females. Per capita recruitment rates were similar for both sexes.

Potentially confounding factors included our relatively small study area size and the presence of a high volume transportation corridor through the middle of our study area. Our study area was approximately five and two times the size of average female and male home ranges respectively. The effects of distance to edge on detection probability would thus have applied to a large percentage of bears in our study. Our SCR models had much better model fit than CR_*dedge*_ and were thus likely to produce more reliable estimates of density and demographic rates. We did not try to differentiate the effects of mortality and emigration on apparent survival [23,49]. However, given our small study area relative to male movements and the low number of observed human-caused mortalities from 2006 through 2008, the decrease in male density was likely caused by emigration and natural mortality. The TransCanada Highway ran through the centre of our study area. Fragmentation effects could have reduced detection probability for traps located on the opposite side of the highway from an individual’s home range centre. While highway crossing structures facilitated breeding [38] and were used by 7 females and 8 males during our study [37] some bears may have avoided the highway and areas with high levels of human use [50]. Future analyses could incorporate the fragmentation effects using spatial capture-recapture models that include measures of habitat quality and connectivity on detection probability [51].

Our averaged grizzly bear densities of 15.1 bears per 1000 km^2^ were similar to densities found in Alberta south of Banff (11.8 to 18.1 bears per 1000 km^2^) and higher than densities found to the north of Banff (4.8 to 5.2 bears per 1000 km^2^) [52]. Our density estimates were much lower than grizzly bear densities found in the Flathead Valley of Southern British Columbia and Glacier Park, Montana (> 30 bears per 1000 km^2^) [53,54] and lower than most regions of British Columbia [55]. Previous studies in Banff tallied the number of radio-collared and unmarked bears to estimate density at between 12 to 16 bears per 1000 km^2^ [56], which was similar to our density. Regional differences in densities were likely driven by ecosystem productivity [55].

The combination of our low density estimates, negative population growth rates, low reproduction rates [48], and higher rates of mortality near people and roads [30,33] suggest that continued grizzly bear monitoring is warranted to determine population trends. Our study design produced parameter estimates with sufficient precision and power to detect changes in density over time. However, expanding the study area both in space and time would help differentiate the relative influence of mortality and emigration on apparent survival, which is important from a management perspective. Moreover, pooling regional DNA-based survey data is desirable to understand landscape scale factors affecting density and population trends. More importantly, continued management actions are necessary to reduce risks of human-caused mortality in Banff and adjacent threatened populations of grizzly bears [52].

Spatial capture-recapture models and to some degree non-spatial capture-recapture models [57–59] are rapidly developing and evolving to better reflect how biological processes, observational processes, study design, habitat quality, and animal movements affect detection probability and density estimates [60–63]. For some species, spatial models can provide more information about biological processes such as survival and emigration versus apparent survival [23,49]. The ability to combine marked, unmarked, and missed individuals identified from multiple sampling approaches such as non-invasive genetic sampling and remote cameras has great potential to substantially improve precision of density estimates [63–65]. Our results suggest that incorporating spatial information into open population capture-recapture models leads to more accurate parameter estimates. Further development in the field of spatial capture-recapture analyses will certainly result in even greater ability to minimize biases and link landscape changes with population dynamics.

## Supporting Information

S1 Data Grizzly bear detection data for Banff National Park from 2006 through 2008. (TXT)

S1 Fig Timing of sampling effort by year and hair detection method. Labels above points indicate the sampling occasion.
(TIFF)

S2 Fig Map of showing the posterior density of grizzly bear activity centres from 2006 through 2008 in Banff National Park, AB, Canada. The spatial distribution of activity centers was influenced by the distribution of traps across the study area and the locations of observed bear detections.
(TIFF)

S1 Table Parameter estimates, 95% HPD intervals, and coefficient of variation from capture-recapture (CR_*dedge*_) and spatial capture-recapture (SCR) models for female, male, and combined sex models. (DOCX)

S2 Table Results from CR, CR_*dedge*_, and SCR open population simulations. (DOCX)

S1 Text JAGS code for CR_*dedge*_ and SCR combined sex grizzly bear open population models. (TXT)

S2 Text R Script for running open population capture-recapture simulations. (TXT)
