# Correction: A Comparison of Grizzly Bear Demographic Parameters Estimated from Non-Spatial and Spatial Open Population Capture-Recapture Models

**DOI:** 10.1371/journal.pone.0137940

**Published:** 2015-09-04

**Authors:** Jesse Whittington, Michael A. Sawaya

There are errors in S2 Fig. Please view the correct S2 Fig below.

## Supporting Information

S2 Fig Map of showing the posterior density of grizzly bear activity centres from 2006 through 2008 in Banff National Park, AB, Canada. The spatial distribution of activity centers was influenced by the distribution of traps across the study area and the locations of observed bear detections.
(TIF)
