# Supplementary figures and images for: A Comparison of Grizzly Bear Demographic Parameters Estimated from Non-Spatial and Spatial Open Population Capture-Recapture Models
Source: PLoS One. 2015 Jul 31;10(7):e0134446. doi: 10.1371/journal.pone.0134446 (PMC4521725; doi:10.1371/journal.pone.0134446)

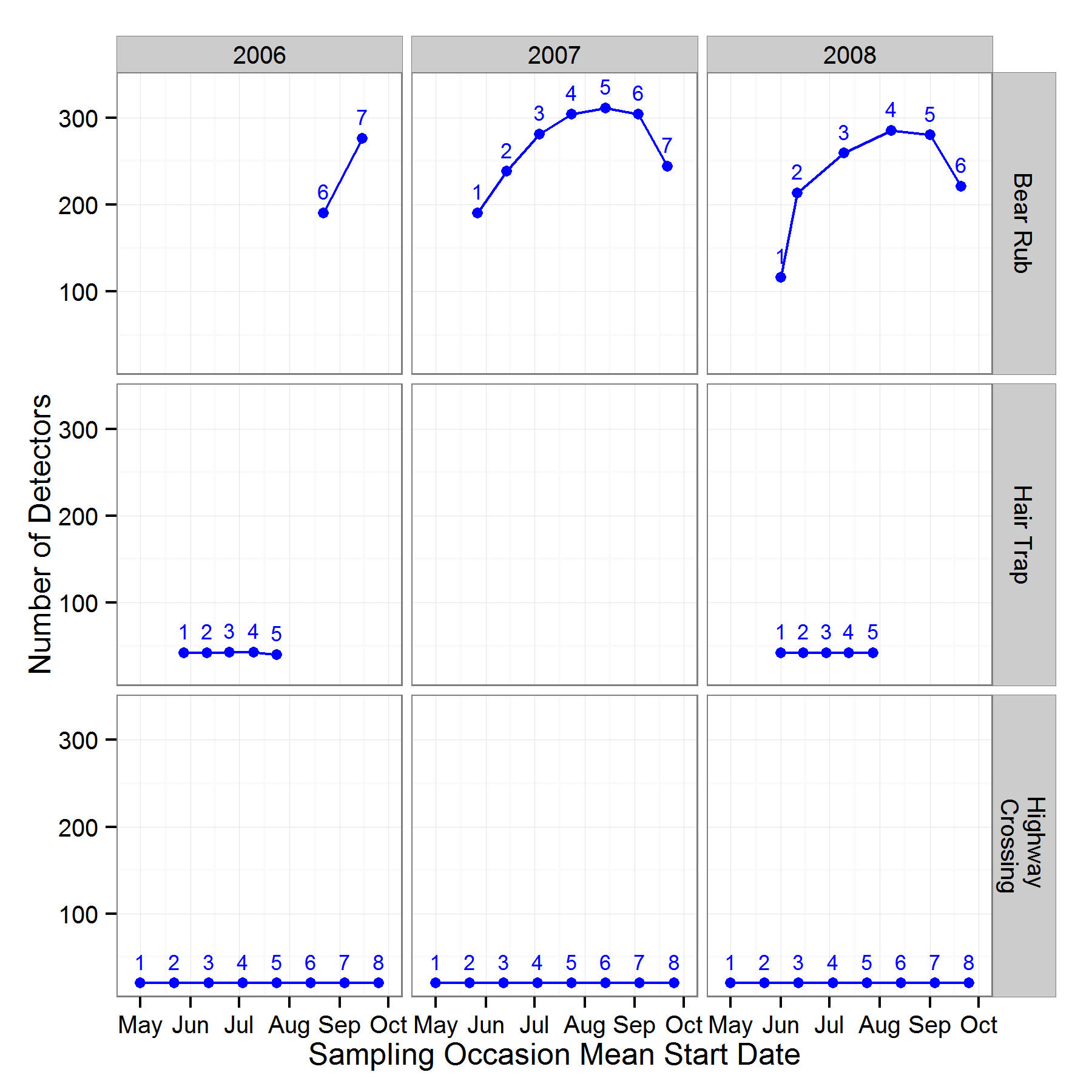

Supplement: S1 Fig — Labels above points indicate the sampling occasion. (TIFF) [file pone.0134446.s002.tiff]

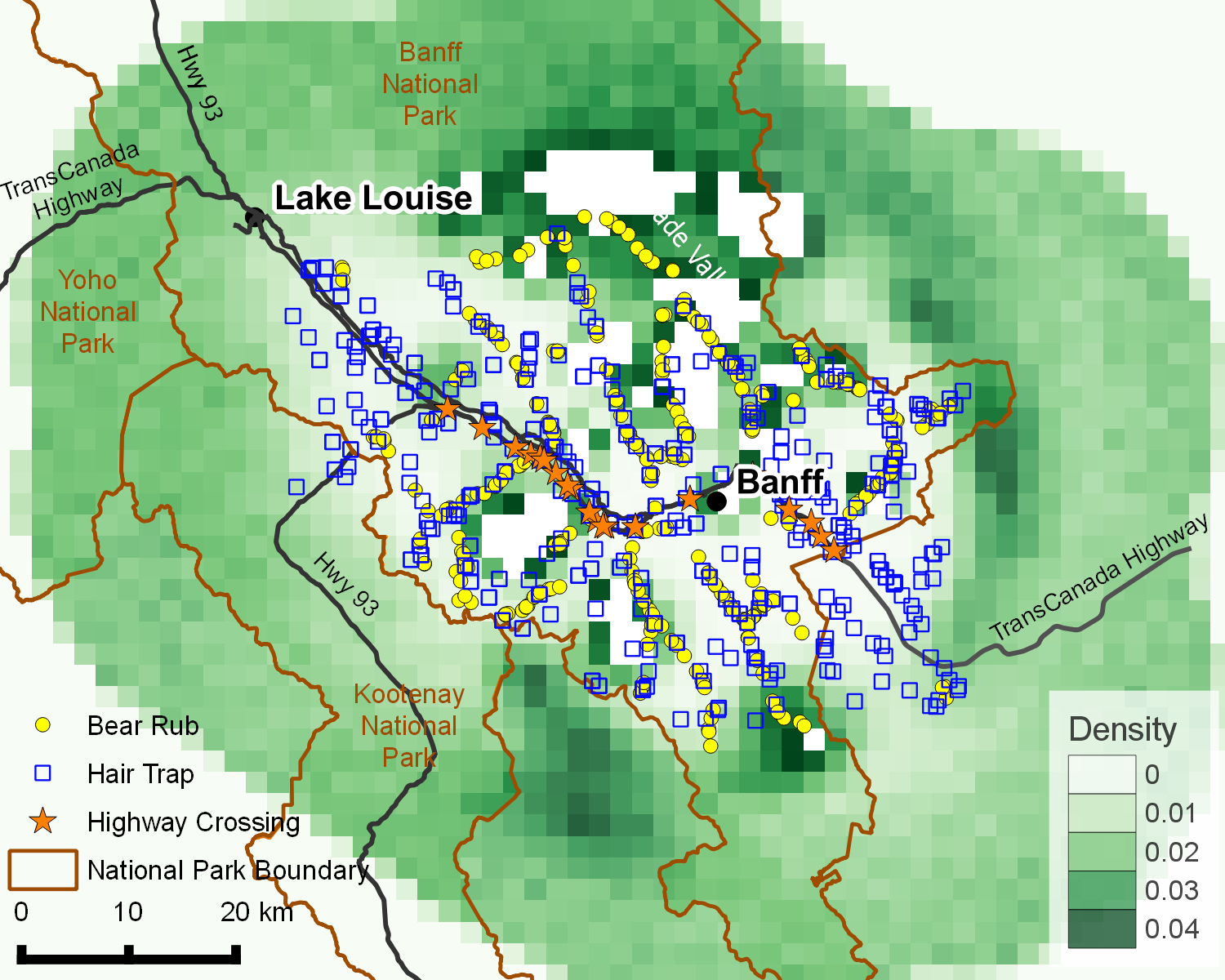

Supplement: S2 Fig — The spatial distribution of activity centers was influenced by the distribution of traps across the study area and the locations of observed bear detections. (TIFF) [file pone.0134446.s003.tiff]
