# Supplementary material for: A Comparison of Grizzly Bear Demographic Parameters Estimated from Non-Spatial and Spatial Open Population Capture-Recapture Models
Source: PLoS One. 2015 Jul 31;10(7):e0134446. doi: 10.1371/journal.pone.0134446 (PMC4521725; doi:10.1371/journal.pone.0134446)
Supplement: S1 Table — (DOCX) [file pone.0134446.s004.docx]

| **Model** | **Sex** | **Parameter** | **Median** | **HPD95.lower** | **HPD95.upper** | **CV** |
| --- | --- | --- | --- | --- | --- | --- |
| CR.dedge | Female | λ_mean_ | 0.894 | 0.758 | 1.024 |  |
| CR.dedge | Female | λ1 | 0.857 | 0.640 | 1.065 |  |
| CR.dedge | Female | λ2 | 0.941 | 0.724 | 1.155 |  |
| CR.dedge | Female | N_mean_ | 28.000 | 24.000 | 33.000 | 7.8 |
| CR.dedge | Female | N1 | 31.000 | 26.000 | 37.000 | 9.8 |
| CR.dedge | Female | N2 | 27.000 | 21.000 | 32.000 | 10.4 |
| CR.dedge | Female | N3 | 26.000 | 22.000 | 30.000 | 8.8 |
| CR.dedge | Female | R_mean_ | 0.076 | 0.000 | 0.152 |  |
| CR.dedge | Female | R1 | 0.091 | 0.023 | 0.207 |  |
| CR.dedge | Female | R2 | 0.074 | 0.000 | 0.227 |  |
| CR.dedge | Female | a.dedge | 0.166 | 0.120 | 0.210 |  |
| CR.dedge | Female | a0.Hair1 | -3.506 | -4.521 | -2.546 |  |
| CR.dedge | Female | a0.Hair2 | -3.177 | -4.086 | -2.261 |  |
| CR.dedge | Female | a0.Hair3 | -1.712 | -2.468 | -0.981 |  |
| CR.dedge | Female | a0.Hair4 | -3.194 | -4.149 | -2.332 |  |
| CR.dedge | Female | a0.Hair5 | -2.004 | -2.771 | -1.233 |  |
| CR.dedge | Female | a0.Hwy1 | -8.194 | -10.000 | -5.865 |  |
| CR.dedge | Female | a0.Hwy2 | -4.668 | -5.957 | -3.534 |  |
| CR.dedge | Female | a0.Hwy3 | -3.693 | -4.608 | -2.813 |  |
| CR.dedge | Female | a0.Hwy4 | -4.182 | -5.239 | -3.195 |  |
| CR.dedge | Female | a0.Hwy5 | -3.690 | -4.600 | -2.812 |  |
| CR.dedge | Female | a0.Hwy6 | -5.496 | -7.301 | -4.008 |  |
| CR.dedge | Female | a0.Hwy7 | -4.402 | -5.542 | -3.334 |  |
| CR.dedge | Female | a0.Hwy8 | -6.370 | -9.011 | -4.450 |  |
| CR.dedge | Female | a0.Rub1 | -7.782 | -10.000 | -5.030 |  |
| CR.dedge | Female | a0.Rub2 | -2.786 | -3.694 | -1.935 |  |
| CR.dedge | Female | a0.Rub3 | -2.912 | -3.813 | -2.057 |  |
| CR.dedge | Female | a0.Rub4 | -2.523 | -3.335 | -1.706 |  |
| CR.dedge | Female | a0.Rub5 | -2.310 | -3.125 | -1.541 |  |
| CR.dedge | Female | a0.Rub6 | -2.179 | -2.908 | -1.471 |  |
| CR.dedge | Female | a0.Rub7 | -3.309 | -4.294 | -2.394 |  |
| CR.dedge | Female | φ_mean_ | 0.803 | 0.673 | 0.920 |  |
| CR.dedge | Female | φ1 | 0.763 | 0.571 | 0.939 |  |
| CR.dedge | Female | φ2 | 0.862 | 0.685 | 0.999 |  |
| CR.dedge | Female | rate.dedge | 0.202 | 0.107 | 0.309 |  |
| CR.dedge | Female | shape.dedge | 1.382 | 0.798 | 2.042 |  |
| CR.dedge | Female | Model Fit | 0.900 | - | - |  |
| CR.dedge | Male | λ_mean_ | 0.825 | 0.700 | 0.948 |  |
| CR.dedge | Male | λ1 | 0.879 | 0.688 | 1.068 |  |
| CR.dedge | Male | λ2 | 0.781 | 0.600 | 0.963 |  |
| CR.dedge | Male | N_mean_ | 36.000 | 33.000 | 40.000 | 4.8 |
| CR.dedge | Male | N1 | 42.000 | 36.000 | 48.000 | 8.0 |
| CR.dedge | Male | N2 | 37.000 | 34.000 | 41.000 | 5.6 |
| CR.dedge | Male | N3 | 29.000 | 26.000 | 33.000 | 7.1 |
| CR.dedge | Male | R_mean_ | 0.112 | 0.039 | 0.193 |  |
| CR.dedge | Male | R1 | 0.100 | 0.000 | 0.216 |  |
| CR.dedge | Male | R2 | 0.132 | 0.071 | 0.200 |  |
| CR.dedge | Male | a.dedge | 0.148 | 0.106 | 0.188 |  |
| CR.dedge | Male | a0.Hair1 | -3.599 | -4.546 | -2.690 |  |
| CR.dedge | Male | a0.Hair2 | -3.598 | -4.557 | -2.703 |  |
| CR.dedge | Male | a0.Hair3 | -3.614 | -4.574 | -2.716 |  |
| CR.dedge | Male | a0.Hair4 | -3.049 | -3.864 | -2.276 |  |
| CR.dedge | Male | a0.Hair5 | -3.566 | -4.518 | -2.656 |  |
| CR.dedge | Male | a0.Hwy1 | -4.085 | -5.029 | -3.211 |  |
| CR.dedge | Male | a0.Hwy2 | -3.678 | -4.506 | -2.892 |  |
| CR.dedge | Male | a0.Hwy3 | -3.796 | -4.639 | -2.966 |  |
| CR.dedge | Male | a0.Hwy4 | -4.091 | -5.044 | -3.218 |  |
| CR.dedge | Male | a0.Hwy5 | -4.266 | -5.262 | -3.308 |  |
| CR.dedge | Male | a0.Hwy6 | -4.723 | -5.981 | -3.671 |  |
| CR.dedge | Male | a0.Hwy7 | -4.728 | -5.949 | -3.646 |  |
| CR.dedge | Male | a0.Hwy8 | -5.538 | -7.313 | -4.074 |  |
| CR.dedge | Male | a0.Rub1 | -1.387 | -2.124 | -0.646 |  |
| CR.dedge | Male | a0.Rub2 | -1.803 | -2.493 | -1.140 |  |
| CR.dedge | Male | a0.Rub3 | -1.712 | -2.387 | -1.053 |  |
| CR.dedge | Male | a0.Rub4 | -1.734 | -2.385 | -1.063 |  |
| CR.dedge | Male | a0.Rub5 | -2.920 | -3.707 | -2.171 |  |
| CR.dedge | Male | a0.Rub6 | -1.780 | -2.381 | -1.193 |  |
| CR.dedge | Male | a0.Rub7 | -1.995 | -2.639 | -1.331 |  |
| CR.dedge | Male | φ_mean_ | 0.708 | 0.590 | 0.822 |  |
| CR.dedge | Male | φ1 | 0.777 | 0.607 | 0.927 |  |
| CR.dedge | Male | φ2 | 0.653 | 0.479 | 0.821 |  |
| CR.dedge | Male | rate.dedge | 0.369 | 0.226 | 0.531 |  |
| CR.dedge | Male | shape.dedge | 2.708 | 1.727 | 3.827 |  |
| CR.dedge | Male | Model Fit | 0.920 | - | - |  |
| CR.dedge | Female + Male | λ_mean_ | 0.863 | 0.771 | 0.957 |  |
| CR.dedge | Female + Male | λ1 | 0.874 | 0.730 | 1.015 |  |
| CR.dedge | Female + Male | λ2 | 0.856 | 0.711 | 1.002 |  |
| CR.dedge | Female + Male | N_mean_ | 64.000 | 59.000 | 69.000 | 4.1 |
| CR.dedge | Female + Male | N1 | 73.000 | 65.000 | 81.000 | 5.9 |
| CR.dedge | Female + Male | N2 | 64.000 | 57.000 | 70.000 | 5.4 |
| CR.dedge | Female + Male | N3 | 55.000 | 50.000 | 61.000 | 5.6 |
| CR.dedge | Female + Male | R_mean_ | 0.100 | 0.047 | 0.153 |  |
| CR.dedge | Female + Male | R1 | 0.091 | 0.024 | 0.176 |  |
| CR.dedge | Female + Male | R2 | 0.115 | 0.055 | 0.197 |  |
| CR.dedge | Female + Male | a.dedge.female | 0.158 | 0.114 | 0.202 |  |
| CR.dedge | Female + Male | a.dedge.male | -0.005 | -0.063 | 0.058 |  |
| CR.dedge | Female + Male | a0.female.Hair1 | -3.402 | -4.391 | -2.450 |  |
| CR.dedge | Female + Male | a0.female.Hair2 | -3.075 | -3.991 | -2.199 |  |
| CR.dedge | Female + Male | a0.female.Hair3 | -1.597 | -2.327 | -0.868 |  |
| CR.dedge | Female + Male | a0.female.Hair4 | -3.084 | -3.986 | -2.182 |  |
| CR.dedge | Female + Male | a0.female.Hair5 | -1.894 | -2.636 | -1.136 |  |
| CR.dedge | Female + Male | a0.female.Hwy1 | -8.163 | -10.000 | -5.794 |  |
| CR.dedge | Female + Male | a0.female.Hwy2 | -4.564 | -5.827 | -3.444 |  |
| CR.dedge | Female + Male | a0.female.Hwy3 | -3.588 | -4.501 | -2.730 |  |
| CR.dedge | Female + Male | a0.female.Hwy4 | -4.079 | -5.158 | -3.099 |  |
| CR.dedge | Female + Male | a0.female.Hwy5 | -3.587 | -4.487 | -2.706 |  |
| CR.dedge | Female + Male | a0.female.Hwy6 | -5.395 | -7.211 | -3.886 |  |
| CR.dedge | Female + Male | a0.female.Hwy7 | -4.295 | -5.427 | -3.239 |  |
| CR.dedge | Female + Male | a0.female.Hwy8 | -6.258 | -8.831 | -4.294 |  |
| CR.dedge | Female + Male | a0.female.Rub1 | -7.697 | -10.000 | -4.933 |  |
| CR.dedge | Female + Male | a0.female.Rub2 | -2.686 | -3.562 | -1.805 |  |
| CR.dedge | Female + Male | a0.female.Rub3 | -2.807 | -3.680 | -1.960 |  |
| CR.dedge | Female + Male | a0.female.Rub4 | -2.414 | -3.231 | -1.621 |  |
| CR.dedge | Female + Male | a0.female.Rub5 | -2.209 | -3.004 | -1.436 |  |
| CR.dedge | Female + Male | a0.female.Rub6 | -2.079 | -2.805 | -1.393 |  |
| CR.dedge | Female + Male | a0.female.Rub7 | -3.209 | -4.185 | -2.295 |  |
| CR.dedge | Female + Male | a0.male.Hair1 | -3.658 | -4.621 | -2.747 |  |
| CR.dedge | Female + Male | a0.male.Hair2 | -3.658 | -4.654 | -2.776 |  |
| CR.dedge | Female + Male | a0.male.Hair3 | -3.678 | -4.644 | -2.783 |  |
| CR.dedge | Female + Male | a0.male.Hair4 | -3.113 | -3.922 | -2.311 |  |
| CR.dedge | Female + Male | a0.male.Hair5 | -3.635 | -4.581 | -2.727 |  |
| CR.dedge | Female + Male | a0.male.Hwy1 | -4.149 | -5.099 | -3.260 |  |
| CR.dedge | Female + Male | a0.male.Hwy2 | -3.732 | -4.556 | -2.936 |  |
| CR.dedge | Female + Male | a0.male.Hwy3 | -3.857 | -4.723 | -3.043 |  |
| CR.dedge | Female + Male | a0.male.Hwy4 | -4.150 | -5.122 | -3.279 |  |
| CR.dedge | Female + Male | a0.male.Hwy5 | -4.323 | -5.336 | -3.391 |  |
| CR.dedge | Female + Male | a0.male.Hwy6 | -4.791 | -5.993 | -3.692 |  |
| CR.dedge | Female + Male | a0.male.Hwy7 | -4.791 | -6.023 | -3.704 |  |
| CR.dedge | Female + Male | a0.male.Hwy8 | -5.608 | -7.419 | -4.154 |  |
| CR.dedge | Female + Male | a0.male.Rub1 | -1.448 | -2.203 | -0.721 |  |
| CR.dedge | Female + Male | a0.male.Rub2 | -1.867 | -2.562 | -1.186 |  |
| CR.dedge | Female + Male | a0.male.Rub3 | -1.781 | -2.455 | -1.128 |  |
| CR.dedge | Female + Male | a0.male.Rub4 | -1.799 | -2.463 | -1.149 |  |
| CR.dedge | Female + Male | a0.male.Rub5 | -2.982 | -3.747 | -2.195 |  |
| CR.dedge | Female + Male | a0.male.Rub6 | -1.839 | -2.441 | -1.252 |  |
| CR.dedge | Female + Male | a0.male.Rub7 | -2.046 | -2.720 | -1.420 |  |
| CR.dedge | Female + Male | p.male | 0.588 | 0.481 | 0.693 |  |
| CR.dedge | Female + Male | φ_mean_ | 0.757 | 0.669 | 0.844 |  |
| CR.dedge | Female + Male | φ1 | 0.781 | 0.652 | 0.898 |  |
| CR.dedge | Female + Male | φ2 | 0.740 | 0.605 | 0.868 |  |
| CR.dedge | Female + Male | rate.dedge | 0.262 | 0.183 | 0.352 |  |
| CR.dedge | Female + Male | shape.dedge | 1.885 | 1.369 | 2.468 |  |
| CR.dedge | Female + Male | Model Fit | 0.970 | - | - |  |
| SCR | Female | D_mean_ | 8.638 | 6.187 | 11.128 | 14.6 |
| SCR | Female | D1 | 9.338 | 6.537 | 12.373 | 16.0 |
| SCR | Female | D2 | 8.404 | 6.070 | 11.556 | 16.7 |
| SCR | Female | D3 | 8.171 | 5.486 | 10.622 | 16.1 |
| SCR | Female | λ_mean_ | 0.925 | 0.786 | 1.071 |  |
| SCR | Female | λ1 | 0.888 | 0.658 | 1.105 |  |
| SCR | Female | λ2 | 0.974 | 0.741 | 1.195 |  |
| SCR | Female | N1 | 80.000 | 56.000 | 106.000 | 16.0 |
| SCR | Female | N2 | 72.000 | 47.000 | 94.000 | 16.7 |
| SCR | Female | N3 | 70.000 | 47.000 | 91.000 | 16.1 |
| SCR | Female | R_mean_ | 0.072 | 0.000 | 0.165 |  |
| SCR | Female | R1 | 0.068 | 0.000 | 0.218 |  |
| SCR | Female | R2 | 0.089 | 0.000 | 0.257 |  |
| SCR | Female | a0.Hair1 | -2.232 | -2.959 | -1.532 |  |
| SCR | Female | a0.Hair2 | -2.305 | -3.043 | -1.640 |  |
| SCR | Female | a0.Hair3 | -1.133 | -1.645 | -0.594 |  |
| SCR | Female | a0.Hair4 | -2.080 | -2.775 | -1.461 |  |
| SCR | Female | a0.Hair5 | -1.342 | -1.890 | -0.818 |  |
| SCR | Female | a0.Hwy1 | -7.601 | -9.999 | -4.769 |  |
| SCR | Female | a0.Hwy2 | -3.372 | -4.510 | -2.356 |  |
| SCR | Female | a0.Hwy3 | -2.149 | -2.800 | -1.487 |  |
| SCR | Female | a0.Hwy4 | -2.760 | -3.601 | -1.971 |  |
| SCR | Female | a0.Hwy5 | -2.473 | -3.238 | -1.762 |  |
| SCR | Female | a0.Hwy6 | -4.170 | -5.953 | -2.821 |  |
| SCR | Female | a0.Hwy7 | -3.127 | -4.162 | -2.230 |  |
| SCR | Female | a0.Hwy8 | -5.050 | -7.730 | -3.173 |  |
| SCR | Female | a0.Rub1 | -8.070 | -10.000 | -5.627 |  |
| SCR | Female | a0.Rub2 | -3.177 | -3.672 | -2.727 |  |
| SCR | Female | a0.Rub3 | -3.179 | -3.626 | -2.725 |  |
| SCR | Female | a0.Rub4 | -3.228 | -3.690 | -2.802 |  |
| SCR | Female | a0.Rub5 | -3.144 | -3.581 | -2.710 |  |
| SCR | Female | a0.Rub6 | -3.196 | -3.614 | -2.814 |  |
| SCR | Female | a0.Rub7 | -3.555 | -4.128 | -3.024 |  |
| SCR | Female | φ_mean_ | 0.837 | 0.712 | 0.948 |  |
| SCR | Female | φ1 | 0.811 | 0.626 | 0.974 |  |
| SCR | Female | φ2 | 0.883 | 0.709 | 1.000 |  |
| SCR | Female | σ | 4.988 | 4.626 | 5.397 |  |
| SCR | Female | Model Fit | 0.520 | - | - |  |
| SCR | Male | D_mean_ | 6.848 | 5.486 | 8.405 | 11.1 |
| SCR | Male | D1 | 7.821 | 5.953 | 9.805 | 13.1 |
| SCR | Male | D2 | 7.120 | 5.720 | 8.988 | 12.2 |
| SCR | Male | D3 | 5.603 | 4.319 | 7.237 | 13.8 |
| SCR | Male | λ_mean_ | 0.844 | 0.703 | 0.975 |  |
| SCR | Male | λ1 | 0.907 | 0.709 | 1.107 |  |
| SCR | Male | λ2 | 0.794 | 0.589 | 0.998 |  |
| SCR | Male | N1 | 67.000 | 51.000 | 84.000 | 13.1 |
| SCR | Male | N2 | 61.000 | 48.000 | 76.000 | 12.2 |
| SCR | Male | N3 | 48.000 | 37.000 | 62.000 | 13.8 |
| SCR | Male | R_mean_ | 0.105 | 0.035 | 0.187 |  |
| SCR | Male | R1 | 0.103 | 0.011 | 0.234 |  |
| SCR | Male | R2 | 0.111 | 0.027 | 0.228 |  |
| SCR | Male | a0.Hair1 | -2.968 | -3.622 | -2.357 |  |
| SCR | Male | a0.Hair2 | -2.966 | -3.645 | -2.339 |  |
| SCR | Male | a0.Hair3 | -3.357 | -4.145 | -2.666 |  |
| SCR | Male | a0.Hair4 | -2.943 | -3.562 | -2.361 |  |
| SCR | Male | a0.Hair5 | -3.284 | -4.068 | -2.567 |  |
| SCR | Male | a0.Hwy1 | -2.752 | -3.510 | -2.045 |  |
| SCR | Male | a0.Hwy2 | -2.174 | -2.777 | -1.584 |  |
| SCR | Male | a0.Hwy3 | -1.965 | -2.555 | -1.427 |  |
| SCR | Male | a0.Hwy4 | -2.745 | -3.513 | -2.050 |  |
| SCR | Male | a0.Hwy5 | -3.023 | -3.885 | -2.264 |  |
| SCR | Male | a0.Hwy6 | -3.407 | -4.438 | -2.517 |  |
| SCR | Male | a0.Hwy7 | -3.647 | -4.758 | -2.623 |  |
| SCR | Male | a0.Hwy8 | -3.650 | -4.776 | -2.644 |  |
| SCR | Male | a0.Rub1 | -1.788 | -2.067 | -1.496 |  |
| SCR | Male | a0.Rub2 | -2.598 | -2.896 | -2.324 |  |
| SCR | Male | a0.Rub3 | -2.918 | -3.226 | -2.613 |  |
| SCR | Male | a0.Rub4 | -3.073 | -3.375 | -2.753 |  |
| SCR | Male | a0.Rub5 | -3.995 | -4.466 | -3.547 |  |
| SCR | Male | a0.Rub6 | -2.847 | -3.122 | -2.589 |  |
| SCR | Male | a0.Rub7 | -2.992 | -3.325 | -2.686 |  |
| SCR | Male | φ_mean_ | 0.730 | 0.606 | 0.843 |  |
| SCR | Male | φ1 | 0.798 | 0.631 | 0.950 |  |
| SCR | Male | φ2 | 0.677 | 0.498 | 0.849 |  |
| SCR | Male | σ | 8.923 | 8.295 | 9.630 |  |
| SCR | Male | Model Fit | 0.450 | - | - |  |
| SCR | Female + Male | D_mean_ | 15.097 | 12.373 | 18.054 | 9.6 |
| SCR | Female + Male | D1 | 16.926 | 13.774 | 20.894 | 10.7 |
| SCR | Female + Male | D2 | 15.175 | 12.140 | 18.560 | 10.8 |
| SCR | Female + Male | D3 | 13.190 | 10.506 | 16.225 | 11.2 |
| SCR | Female + Male | λ_mean_ | 0.882 | 0.779 | 0.981 |  |
| SCR | Female + Male | λ1 | 0.896 | 0.743 | 1.042 |  |
| SCR | Female + Male | λ2 | 0.871 | 0.713 | 1.028 |  |
| SCR | Female + Male | N1 | 145.000 | 113.000 | 174.000 | 10.7 |
| SCR | Female + Male | N2 | 130.000 | 102.000 | 157.000 | 10.8 |
| SCR | Female + Male | N3 | 113.000 | 89.000 | 138.000 | 11.2 |
| SCR | Female + Male | R_mean_ | 0.087 | 0.032 | 0.152 |  |
| SCR | Female + Male | R1 | 0.080 | 0.011 | 0.175 |  |
| SCR | Female + Male | R2 | 0.101 | 0.025 | 0.200 |  |
| SCR | Female + Male | a0.female.Hair1 | -2.220 | -2.955 | -1.523 |  |
| SCR | Female + Male | a0.female.Hair2 | -2.296 | -3.030 | -1.606 |  |
| SCR | Female + Male | a0.female.Hair3 | -1.119 | -1.665 | -0.602 |  |
| SCR | Female + Male | a0.female.Hair4 | -2.075 | -2.736 | -1.438 |  |
| SCR | Female + Male | a0.female.Hair5 | -1.335 | -1.858 | -0.799 |  |
| SCR | Female + Male | a0.female.Hwy1 | -7.585 | -9.999 | -4.728 |  |
| SCR | Female + Male | a0.female.Hwy2 | -3.377 | -4.536 | -2.393 |  |
| SCR | Female + Male | a0.female.Hwy3 | -2.150 | -2.829 | -1.509 |  |
| SCR | Female + Male | a0.female.Hwy4 | -2.750 | -3.598 | -1.956 |  |
| SCR | Female + Male | a0.female.Hwy5 | -2.478 | -3.216 | -1.756 |  |
| SCR | Female + Male | a0.female.Hwy6 | -4.160 | -5.940 | -2.833 |  |
| SCR | Female + Male | a0.female.Hwy7 | -3.129 | -4.135 | -2.233 |  |
| SCR | Female + Male | a0.female.Hwy8 | -5.051 | -7.890 | -3.179 |  |
| SCR | Female + Male | a0.female.Rub1 | -8.085 | -10.000 | -5.637 |  |
| SCR | Female + Male | a0.female.Rub2 | -3.171 | -3.651 | -2.705 |  |
| SCR | Female + Male | a0.female.Rub3 | -3.178 | -3.641 | -2.736 |  |
| SCR | Female + Male | a0.female.Rub4 | -3.228 | -3.698 | -2.795 |  |
| SCR | Female + Male | a0.female.Rub5 | -3.144 | -3.597 | -2.719 |  |
| SCR | Female + Male | a0.female.Rub6 | -3.193 | -3.584 | -2.782 |  |
| SCR | Female + Male | a0.female.Rub7 | -3.557 | -4.129 | -3.036 |  |
| SCR | Female + Male | a0.male.Hair1 | -2.968 | -3.656 | -2.370 |  |
| SCR | Female + Male | a0.male.Hair2 | -2.964 | -3.608 | -2.342 |  |
| SCR | Female + Male | a0.male.Hair3 | -3.353 | -4.138 | -2.656 |  |
| SCR | Female + Male | a0.male.Hair4 | -2.941 | -3.565 | -2.338 |  |
| SCR | Female + Male | a0.male.Hair5 | -3.290 | -4.074 | -2.611 |  |
| SCR | Female + Male | a0.male.Hwy1 | -2.742 | -3.486 | -2.031 |  |
| SCR | Female + Male | a0.male.Hwy2 | -2.168 | -2.795 | -1.589 |  |
| SCR | Female + Male | a0.male.Hwy3 | -1.958 | -2.530 | -1.403 |  |
| SCR | Female + Male | a0.male.Hwy4 | -2.738 | -3.500 | -2.049 |  |
| SCR | Female + Male | a0.male.Hwy5 | -3.020 | -3.892 | -2.244 |  |
| SCR | Female + Male | a0.male.Hwy6 | -3.407 | -4.438 | -2.507 |  |
| SCR | Female + Male | a0.male.Hwy7 | -3.645 | -4.776 | -2.659 |  |
| SCR | Female + Male | a0.male.Hwy8 | -3.635 | -4.782 | -2.663 |  |
| SCR | Female + Male | a0.male.Rub1 | -1.783 | -2.068 | -1.499 |  |
| SCR | Female + Male | a0.male.Rub2 | -2.596 | -2.886 | -2.314 |  |
| SCR | Female + Male | a0.male.Rub3 | -2.916 | -3.241 | -2.620 |  |
| SCR | Female + Male | a0.male.Rub4 | -3.070 | -3.384 | -2.765 |  |
| SCR | Female + Male | a0.male.Rub5 | -3.993 | -4.477 | -3.557 |  |
| SCR | Female + Male | a0.male.Rub6 | -2.846 | -3.115 | -2.592 |  |
| SCR | Female + Male | a0.male.Rub7 | -2.990 | -3.322 | -2.677 |  |
| SCR | Female + Male | p.male | 0.459 | 0.350 | 0.573 |  |
| SCR | Female + Male | φ_mean_ | 0.787 | 0.697 | 0.871 |  |
| SCR | Female + Male | φ1 | 0.814 | 0.686 | 0.929 |  |
| SCR | Female + Male | φ2 | 0.769 | 0.635 | 0.892 |  |
| SCR | Female + Male | σ female | 4.993 | 4.615 | 5.382 |  |
| SCR | Female + Male | σ male | 8.964 | 8.309 | 9.641 |  |
| SCR | Female + Male | Model Fit | 0.470 | - | - |  |

Parameter descriptions: φ = apparent survival, R = per capita recruitment, λ = population growth rate, N = number of individuals, D = density per 1000 km^2^, σ = the scale parameter for detection probability, a0 = occasion specific detection probability by trap type on the logit scale, a.dedge = coefficient for the effect of distance to edge on detection probability on the logit scale, rate.dedge and shape.dedge were estimates for parameter estimates for the density and distribution of distance to edge with a gamma distribution, Model Fit = Bayesian P-value where values < 0.05 or > 0.95 indicate poor fit.
