# Supplementary material for: A Comparison of Grizzly Bear Demographic Parameters Estimated from Non-Spatial and Spatial Open Population Capture-Recapture Models
Source: PLoS One. 2015 Jul 31;10(7):e0134446. doi: 10.1371/journal.pone.0134446 (PMC4521725; doi:10.1371/journal.pone.0134446)
Supplement: S2 Table — (DOCX) [file pone.0134446.s005.docx]

Table S2. Average posterior bias, SE of medians, credible interval coverage, and power from 100 simulated data sets analysed and presented in order of capture - recapture, capture - recapture with distance to edge, and spatial capture - recapture models.

| **Parameter** | **D** | **g0** | **TrueValue** | **Bias** | **SE.Median** | **CIC** | **Power** |
| --- | --- | --- | --- | --- | --- | --- | --- |
| Lambda.R | 0.5 | 0.1 | 0.9 | -0.00/-0.01/0.00 | 0.08/0.08/0.06 | 88/89/96 | 49/45/35 |
| Lambda.R | 0.5 | 0.5 | 0.9 | -0.01/-0.01/-0.01 | 0.06/0.05/0.05 | 86/86/91 | 70/71/64 |
| Lambda.R | 1.0 | 0.1 | 0.9 | 0.00/-0.01/0.00 | 0.06/0.06/0.05 | 89/91/96 | 61/68/59 |
| Lambda.R | 1.0 | 0.5 | 0.9 | -0.00/-0.01/-0.00 | 0.04/0.04/0.03 | 87/87/94 | 84/88/87 |
| phi | 0.5 | 0.1 | 0.8 | -0.06/-0.03/-0.01 | 0.06/0.07/0.06 | 84/89/94 |  |
| phi | 0.5 | 0.5 | 0.8 | -0.05/-0.03/-0.01 | 0.05/0.05/0.05 | 88/94/96 |  |
| phi | 1.0 | 0.1 | 0.8 | -0.06/-0.03/-0.01 | 0.05/0.05/0.04 | 70/88/94 |  |
| phi | 1.0 | 0.5 | 0.8 | -0.03/-0.01/0.00 | 0.03/0.03/0.03 | 82/98/94 |  |
| R | 0.5 | 0.1 | 0.1 | 0.06/0.03/0.01 | 0.06/0.05/0.05 | 60/78/92 |  |
| R | 0.5 | 0.5 | 0.1 | 0.04/0.02/0.00 | 0.04/0.04/0.04 | 2/48/72 |  |
| R | 1.0 | 0.1 | 0.1 | 0.06/0.02/0.01 | 0.05/0.04/0.04 | 42/77/86 |  |
| R | 1.0 | 0.5 | 0.1 | 0.03/0.00/-0.00 | 0.03/0.03/0.02 | 4/55/71 |  |
| sigma | 0.5 | 0.1 | 1.0 | na/na/0.00 | na/na/0.03 | na/na/94 |  |
| sigma | 0.5 | 0.5 | 1.0 | na/na/-0.00 | na/na/0.01 | na/na/97 |  |
| sigma | 1.0 | 0.1 | 1.0 | na/na/0.00 | na/na/0.02 | na/na/95 |  |
| sigma | 1.0 | 0.5 | 1.0 | na/na/-0.00 | na/na/0.01 | na/na/96 |  |
