# Supplementary figures and images for: Correction: A Comparison of Grizzly Bear Demographic Parameters Estimated from Non-Spatial and Spatial Open Population Capture-Recapture Models
Source: PLoS One. 2015 Sep 4;10(9):e0137940. doi: 10.1371/journal.pone.0137940 (PMC4560396; doi:10.1371/journal.pone.0137940)

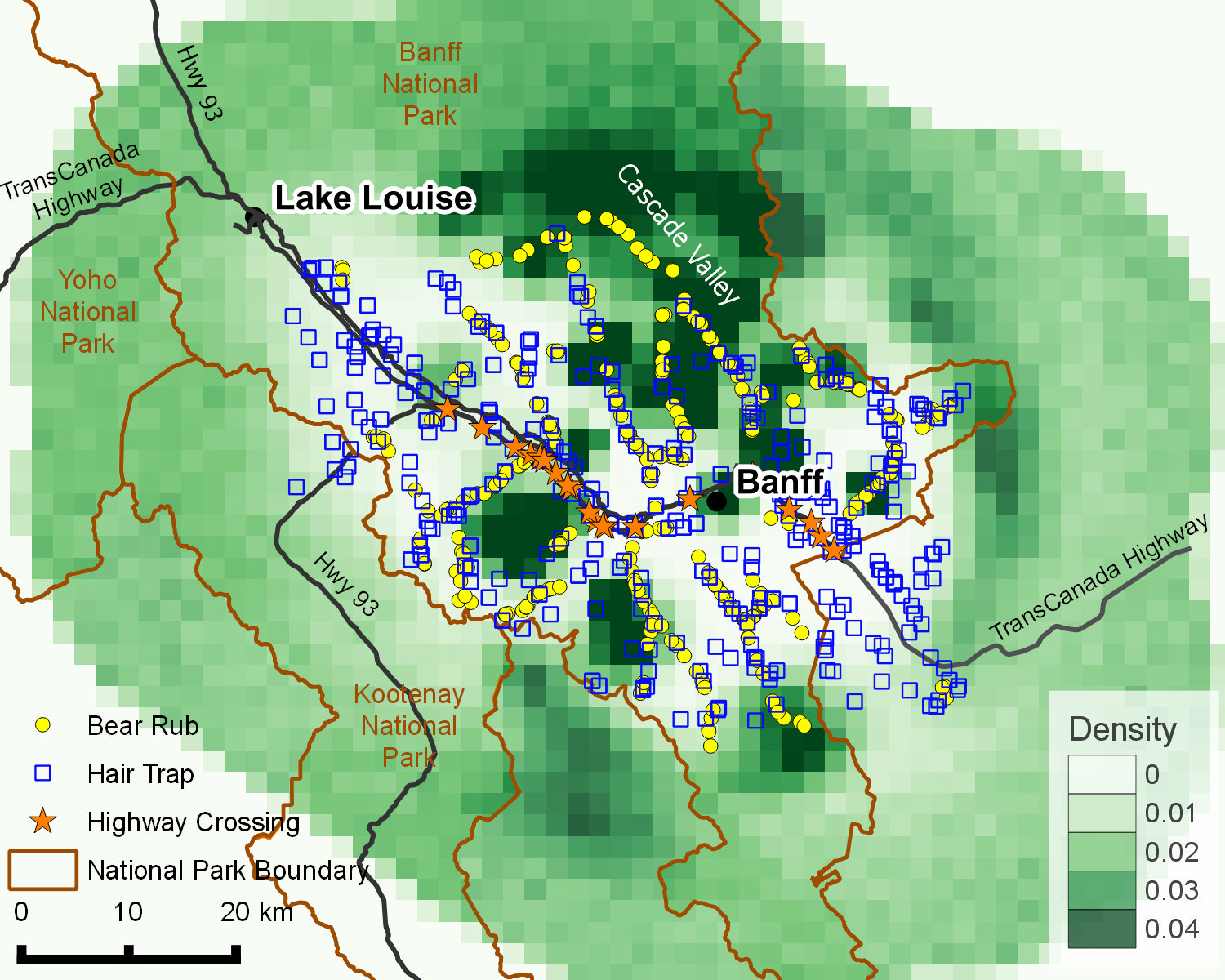

Supplement: S2 Fig — The spatial distribution of activity centers was influenced by the distribution of traps across the study area and the locations of observed bear detections. (TIF) [file pone.0137940.s001.tif]
